# Reality Check: The Aspirations of the European Health Data Space Amidst Challenges in Decentralized Data Analysis

**DOI:** 10.2196/76491

**Published:** 2025-09-19

**Authors:** Holger Fröhlich, Anne Funck Hansen, Mika Hilvo, Gunther Jansen, Sumit Madan, Sobhan Moazemi, Sanziana Negreanu, Venkata Satagopam, Phil Gribbon, Christian Muehlendyck

**Affiliations:** 1 Fraunhofer Institute for Algorithms and Scientific Computing, Schloss Birlinghoven, Sankt Augustin, 53757, Germany, 49 151 7059794; 2 Fraunhofer Society for the Advancement of Applied Research, Munich, Germany; 3 VTT Technical Research Centre of Finland, Espoo, Finland; 4 Novartis (Switzerland), Basel, Switzerland; 5 Johnson & Johnson (Germany), Norderstedt, Germany; 6 Luxembourg Centre for Systems Biomedicine, Belvaux, Luxembourg; 7 Fraunhofer Institute for Translational Medicine and Pharmacology, Hamburg, Germany

**Keywords:** European Health Data Space, federated learning, swarm learning, General Data Protection Rule, federated data analysis

## Abstract

The European Health Data Space (EHDS) aspires to enable secure, interoperable, and decentralized health data usage across Europe. This paper explores legal and technical challenges in implementing EHDS goals, particularly for secondary data use. It highlights federated and swarm learning as promising yet complex solutions, requiring robust infrastructure, standardization, and regulatory clarity. We emphasize the need for coordinated legislative and technological advances to realize EHDS ambitions.

## Introduction

### Background

The European Health Data Space (EHDS) aims at transforming health care delivery, innovation, and research across Europe [1]. The EHDS is based on a legal framework on which the European Parliament and the Council reached a political agreement in spring 2024. It has the following three overarching goals: control over personal health data, developing a market for electronic health records (EHRs), and facilitating secondary data use.

### Control Over Personal Health Data

This goal is to ensure that individuals can access and manage their own health data securely, which is at the core of the EHDS vision. By enabling portability of data across the European Union (EU), the initiative seeks to empower the citizens and health care providers to make better-informed decisions. Notably, member states can offer a complete drop-out to their citizens.

### Developing a Market for EHRs

The EHDS seeks to initiate a competitive market for EHR systems that are accessible, efficient, secure, and interoperable across member states.

### Facilitating Secondary Data Use

The EHDS aims to not only break down silos that hinder the flow of data between stakeholders in the health care system, but also promote the secondary use of these data for research, innovation, policy-making, and regulatory activities.

The EHDS primary legislation provides an overarching framework but does not provide any technical infrastructure or implementation details [2]. However, the secondary legislation and guidance documents from ongoing initiatives such as TEHDAS2 [3] are working on further details to support the implementation in the EU member states.

## Challenges

**Figure 1.**
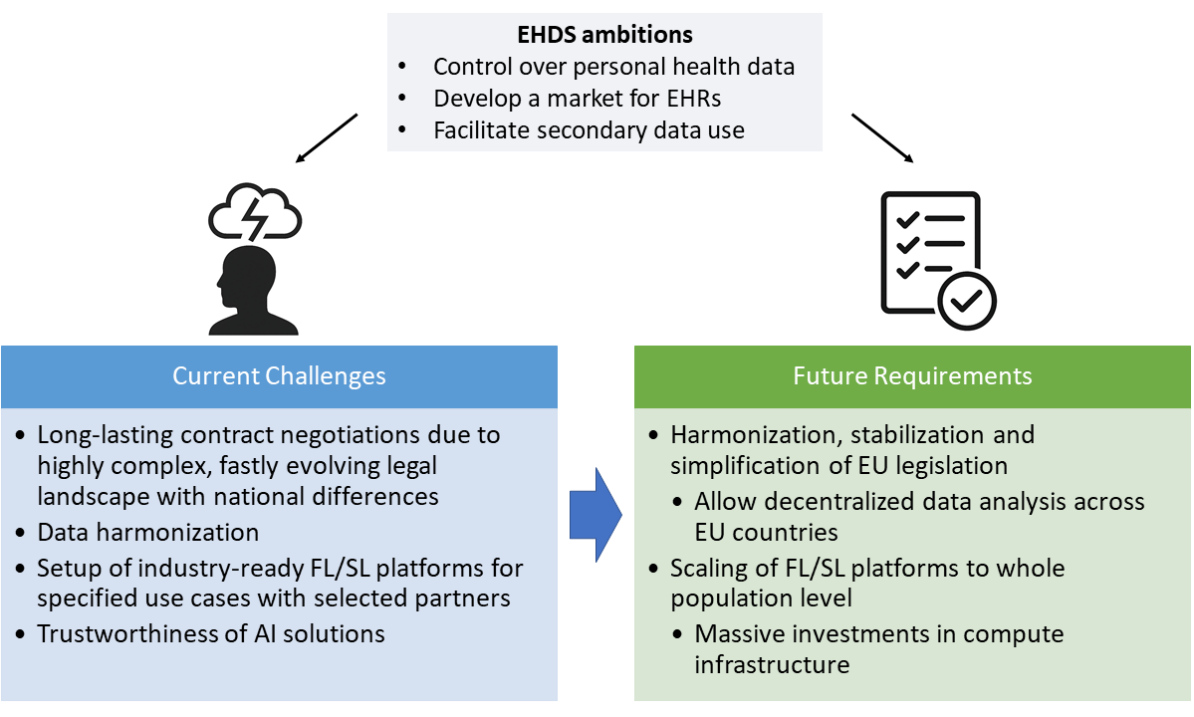
Current challenges and future requirements for a successful realization of the European Health Data Space (EHDS). AI: artificial intelligence; EHR: electronic health record; EU: European Union; FL: federated machine learning; SL: swarm learning.

Projects aiming at implementing a technical infrastructure compatible with the secondary legislation ambitions of the EHDS are currently confronted with several legal as well as technical challenges (Figure 1).

### Legal Challenges

The ambition of the EHDS to establish a market for EHRs and to facilitate the secondary use of health data is challenging considering the current heterogeneous interpretation of the General Data Protection Regulation (GDPR) regarding the use of sensitive patient-level data [4]. While processing of health data without the explicit consent of patients is principally possible for research purposes within the GDPR, this typically requires careful documentation, risk assessments, and compliance agreements, all of which pose significant challenges to collaborative research initiatives. Moreover, ethical board approvals, based on detailed study protocols, are often necessary. Altogether, fulfilling all legal and ethical requirements can easily last 1 to‐2 years in practice.

Subsequently, access to data often further necessitates additional agreements defining the terms of, eg, costs, split of intellectual property rights, and liability, which require additional time, effort, and trust between parties. Moreover, national variations exist in the implementation of the GDPR and the requirements for ethical board approvals throughout the EU. Overall, the current legal landscape provides robust protection for patient data; however, its complexity complicates and slows down innovation projects, and problems multiply with the number of involved parties, sectors, and jurisdictions.

### Technical Challenges

From a technical point of view, differences in data formats and standards are further hurdles to realize the requested portability of health data across the EU. Hence, initiatives like the Observational Medical Outcomes Partnership (OMOP) aim at establishing a common data model (CDM) designed to standardize the structure and content of observational health data [5]. In consequence, multiple initiatives (eg, EHDEN [6] and DARWIN-EU [7]) have been initiated to map real-world data collected during routine health care to OMOP and to develop the corresponding harmonization processes [8]. These initiatives thus address semantic interoperability of health data as a prerequisite for portability. However, data harmonization processes are not only complex, time consuming, and error prone, but also raise the question of how to deal with valuable non-standard data elements, which may only exist at national or institutional levels. The EHDS could amplify the impact of data harmonization initiatives through the adoption of standards and technical requirements across EU countries.

## Decentralized Data Analysis as an Enabling Technology

### Background

Decentralized data analytical approaches, including federated machine learning (FL) [9], offer a solution to overcome legal and technical challenges by enabling collaborative data analysis without necessitating centralized data storage. In FL, models are trained locally on data held by individual organizations. Only the model updates, not the raw data, are shared with a central server, which orchestrates the training process. FL enhances privacy by keeping sensitive data on-premises while enabling collaborative model training. Swarm learning (SL) [10] is a FL variant that removes the need for a central coordinating server. Instead, a blockchain is used to ensure direct and secure communication between local servers. SL offers improved scalability and faster model convergence compared to traditional FL, because it supports asynchronous learning and thus reduces the communication bottleneck with a central server. Major commercial players today provide robust libraries for federated data analysis, including descriptive summary statistics and FL/SL, thus lowering the barrier to employing such technologies in practice. Projects aiming for the implementation of EHDS-compatible platforms can thus build on relatively mature technology here.

### Concerns in Decentralized Data Analysis

Although decentralized data analysis reduces the need for data transfer, it is not immune to risks. For example, machine learning (ML) model updates can inadvertently reveal sensitive information through reverse engineering or gradient leakage [11]. Consequently, approaches such as differential privacy and encryption technologies remain essential safeguards. Differential privacy amounts to adding a defined amount of noise to model gradients during local model updates. However, this can degrade model performance and can only be mitigated by prolonged model training, which often proves computationally impractical.

Another challenge arises from the propagation of biases inherent in local datasets to the aggregated global model. This issue can be intensified when larger datasets have a disproportional influence. Furthermore, variations in data quality and heterogeneity across sites can affect ML model performance, necessitating rigorous validation and harmonization efforts across all parties participating in a decentralized data analysis. At the same time, the identification of possible reasons for biases in an ML model or generally poor prediction performance is extremely hard to detect if data scientists have no direct access to data.

While recent techniques allow the provision of human-understandable explanations of predictions produced by neural networks and can thus help to identify possibly model biases [12-14], these methods are not guaranteed to conform between models trained on different local and pooled datasets [11]. FL/SL approaches are thus in a certain tension with the general ambition of trustworthiness of AI formulated by the EU [15].

### Technical Requirements

On a technical level, decentralized data analysis, including FL/SL, requires setting up a cloud computing infrastructure. For this purpose, multiple commercial platforms are now available. In addition, European projects such as IDERHA (Integration of Heterogeneous Data and Evidence towards Regulatory and HTA Acceptance [16]) aim for setting up a scalable cloud computing infrastructure with security measures that meet the requirements of the European health care sector. Still, there is a need to critically evaluate those solutions regarding computational infrastructure (eg, support of GPU usage), security of communication between organizations, authentication of users, and support of FL/SL. Notably, the latter may require opening specific ports at each of the participating sites, which in turn may only be possible on specific servers that are hosted in a demilitarized zone. Finally, interoperability is key. Hence, it is essential to map data at each participating organization to a standardized CDM such as OMOP.

### Legal Requirements

The legal challenges outlined above necessitate that research projects address technical and legal data governance from the outset, as they are integral components of improving access to and secondary use of health data. Effective legal facilitation of data-driven health innovation must ensure robust protection of data subject rights while mitigating compliance risks for involved entities. Simultaneously, the legal governance framework should be designed to minimize overregulation and redundancy, as such issues directly undermine the workability and impact of the technical solutions it aims to support. Given the complexity of the current regulatory and technical landscape, a scalable and modular governance approach is essential. IDERHA has developed and successfully piloted such a data governance model, which has already been adopted by several other research initiatives, such as CERTAINTY [17].

## Existing Example Projects

As of January 2025, the CORDIS web portal of the EU lists 14 projects in the health care domain after using the search terms “federated learning,” “swarm learning,” and “federated machine learning” with an OR conjunction. Following manual inspection, 8 of them mention FL in the description of their objectives. Among those 8 projects (AI-SPRINT, dAIbetes, UMBRELLA, NextGen, SEARCH, DTRIP4H, INCISIVE, and ORCHESTRA), two (INCISIVE and ORCHESTRA) have recently been completed. This shows the topicality of FL/SL concepts while at the same time highlighting that most applications of FL/SL in health care are still in an early exploratory phase.

INCISIVE [18] delivered a federated data analysis platform for artificial intelligence (AI)–based diagnosis of different cancers (breast, lung, colorectal, and prostate cancers), mostly focusing on medical images. After registration, the user can search through the available data and train AI/ML models. Moreover, organizations can contribute their own data and become a member of the cloud infrastructure.

ORCHESTRA [19] focused on the compilation and federated analysis of an EU-wide cohort to support research on SARS-CoV-2. Users can get a high-level overview about the studies included, including a standardized list of variables. Furthermore, they can apply to access specific studies.

The German Center for Neurodegenerative Diseases (DZNE) currently uses SL in the context of the early detection of Alzheimer disease, Parkinson disease, COVID-19, long COVID syndromes, leukemia, and infectious diseases [20].

Hussein et al [2] outline additional initiatives, and further results are anticipated in the coming years as ongoing projects conclude.

While this discussion mainly focuses on the connection of FL/SL with the ambitions of the EHDS, it is important to mention that FL/SL platforms are also developed in other regions of the world. For example, the Mayo clinics in the USA launched an FL platform to predict response to chemotherapy [21]. The Intel Labs and the Medical School of the University of Pennsylvania initiated a large FL study involving medical imaging data from 71 sites across six continents [22]. In addition, commercial players are now offering FL platforms.

## Challenges for Translation Into Market-Ready Solutions

The discussed examples show the first successful implementations of decentralized data analysis as proof of concept in research. Nevertheless, we expect a long way to go until market-ready high-performance infrastructures for the analysis of health data at whole population levels across Europe are available. At this point, specifically, FL/SL generates internet traffic, which increases with the number of model parameters. This can significantly slow down computation and thus negatively impact computationally intensive AI/ML applications.

Some EU countries allow the analysis of EHRs from national health registries only within authenticated environments. For instance, in Finland, health data for secondary use must be processed in secure processing environments that do not allow analysis results or model parameters to automatically leave or enter the environment. Currently, this prevents Finnish participation in a decentralized data analysis infrastructure. The EHDS aims for data access and processing within secure processing environments provided by health data access bodies. To enable the vision of the EHDS, such secure processing environments must in the future allow the use of decentralized data analysis, including FL/SL.

Further challenges arise as soon as AI/ML models should be deployed for routine health care, which is essential for generating real benefit for patients. AI/ML models used in health care must comply with the Medical Device Regulation [23], which mandates rigorous validation and monitoring processes to ensure safety and efficacy. Furthermore, the EU AI Act classifies health care–related AI/ML models as high-risk systems [24]. This imposes additional requirements for transparency, accountability, and risk management, increasing the burden on developers.

## Conclusions

The EHDS represents a bold vision for the future of health care in Europe, including control over personal health data, development of a market for EHRs, and facilitating secondary data use. These ambitions have to be balanced against concerns regarding data privacy and compliance with regulatory frameworks.

Although decentralized data analysis, including FL/SL, is not free of technical concerns and despite the limitation of trustworthiness of AI solutions, decentralized data analysis offers a promising solution to these legal challenges. However, its implementation requires significant investment in scalable technical infrastructure, data standardization, and data governance. While first example projects and commercial solutions for specific use cases and with selected partner organizations exist, the application of FL/SL in health care is generally in an early, exploratory phase. Scaling existing prototypes to enable whole population data analysis will require massive investments in technical infrastructure. In addition, deployment of AI/ML models trained in a decentralized manner for routine health care comes with additional technical, practical, and regulatory challenges.

Currently, long-lasting legal and regulatory processes for the setup of decentralized data analysis platforms cannot be circumvented, and differences in national legislation across EU member states prevent an equitable participation in decentralized data analysis initiatives. This situation can be viewed as a competitive disadvantage compared to other areas of the world. While scientific progress will likely reduce some of the technical constraints, decision makers should think about the possibilities to strengthen the predictability and coherence of the EU and national legislations stemming from the EU Digital Health and the EU Digitalization Strategies. Organizations must navigate an increasingly complex and quickly evolving legal landscape, in which they have to comply with the GDPR, the EHDS, and the AI Act in the future. While projects like IDERHA have come up with constructs for sharing data in compliance with the current legislation, the impact of upcoming legislations remains to be determined. Altogether, there is a strong need for a better harmonization, stabilization, and simplification of the EU legislation to make the EHDS a success story. In this regard, the decentralized analysis of health data across EU countries has to be legally enabled.

In conclusion, there is a long way to go before the EHDS can deliver on its promise of a better connected and efficient health ecosystem across Europe.

## Abbreviations

AI: artificial intelligence
CDM: common data model
DZNE: German Center for Neurodegenerative Diseases
EHDS: European Health Data Space
EHR: electronic health record
EU: European Union
FL: federated machine learning
GDPR: General Data Protection Regulation
IDERHA: Integration of Heterogeneous Data and Evidence towards Regulatory and HTA Acceptance
ML: machine learning
OMOP: Observational Medical Outcomes Partnership
SL: swarm learning
